# Putative identification of CASTOR1 as one of the targets of ganoderic acid a via thermal proteome profiling and molecular docking

**DOI:** 10.3389/fphar.2026.1666269

**Published:** 2026-04-08

**Authors:** Aifang Chen, Weishuai Xu, Yaxin Wang, Hualun Liang, Diling Chen

**Affiliations:** 1 GuangDong Provincial Hospital of Chinese Medicine, Guangzhou, Guangdong, China; 2 Guangdong Yier Biotechnology Limited Company, Guangzhou, Guangdong, China

**Keywords:** ganoderic acid A, gene ontology (GO), kyoto encyclopedia ofGenes and genomes(KEGG), molecular docking, thermal proteome profiling

## Abstract

In traditional Chinese medicine theory, aging is hypothesized to arise from severe deficiency of kidney essence, which is posited to induce “emptiness of the sea of marrow”. Ganoderma has been documented to possess properties that nourish kidney qi and enrich essence and blood. It has been demonstrated that ganoderma triterpenoids are capable of mitigating age-related cerebral atrophy and retarding the aging process in murine models. Ganoderic acid A (GAA) has been characterized as the principal bioactive metabolite of these triterpenoids. Leveraging thermal proteomics profiling, CASTOR1 was identified as a target protein exhibiting significant disparities in melting curves. Through functional annotation via GO terms and pathway analysis using KEGG, it has been indicated that GAA may modulate metabolic regulatory pathways through interaction with specific molecular targets. A pronounced association between GAA and the GATOR2 complex, a downstream effector of CASTOR1, has been revealed by GO enrichment analysis, suggesting a potential mechanistic link. Thus, it is inferred that one potential target of GAA in neuronal cells is presumably CASTOR1 protein. It is hypothesized that ganoderic acid A exerts its pharmacological effects likely through regulation mediated by the potential target protein CASTOR1, which in turn modulates the mTOR signaling pathway.

## Background

*Ganoderma lucidum* has been designated as the “Divine Mushroom,” “Elixir of Immortality,” and “Eternal Youth botanical drug” in canonical texts such as Shennong’s botanical drug Classic, where it was categorized as a superior-grade botanical drug with properties to “tonify the middle burner, augment intellect, and arrest forgetfulness.” Prolonged administration was documented to mitigate physical debilitation, retard senescence, and extend lifespan (Fang et al., 2025). Within traditional medicine frameworks, consistent ingestion of Ganoderma is posited to confer longevity and “fortify vital qi to consolidate constitutional integrity.”

The doctrine that “the kidney governs bone marrow production, which communicates with the brain” is elaborated in The Yellow Emperor’s Inner Canon, thereby establishing the physiological nexus between renal essence and cerebral function. Characteristic neuropathological hallmarks in Alzheimer’s disease (AD) patients, including cortical atrophy, sulcal widening, and a 20% reduction in encephalic mass, have been disclosed by anatomical investigations (Roe et al., 2022; Wang et al., 2025). This constellation of findings is denominated “marrow sea emptiness” in traditional Chinese medicine (TCM), a condition ascribed to profound depletion of renal essence in geriatric populations, which culminates in insufficient nourishment of cerebral marrow and subsequent cognitive regression (Yang et al., 2024; Qi Y et al., 2024). Accordingly, the therapeutic tenet for addressing cerebral aging in TCM is centered on replenishing renal essence to engender marrow and nourish the brain. Ganoderma is acknowledged in TCM as sweet in flavor and neutral in nature, acting upon the heart, lung, liver, and kidney meridians to nourish renal qi, enrich blood essence, regulate renal function, ameliorate cognitive performance, and pacify the psyche (Qiao et al., 2025; Zeng et al., 2021). Its efficacy in augmenting immune competence, restoring metabolic equilibrium (Qi Z et al., 2024), neutralizing oxidative stressors (Zhu et al., 2013), facilitating nuclear DNA biosynthesis (Kang et al., 2019), impeding neoplastic transformation (Li L et al., 2019), alleviating age-related pathophysiology, preserving hepatic homeostasis, and modulating lipid/glucose dynamics has been corroborated by contemporary scientific inquiries.

Ganoderma extracts are characterized by complex chemical compositions, among which polysaccharides and Ganoderma triterpenoids are recognized as the primary pharmacologically active metabolites (Nie et al., 2025). Modern studies have revealed that Ganoderma triterpenoids are closely implicated in the progression of organismal aging. Ganoderma triterpenoids are proposed to attenuate physiological brain degeneration and ameliorate pathological manifestations such as hair loss and skin sagging in aging mice, effects mediated by the regulation of sphingolipid metabolism, telomere elongation, enhanced autophagy, and the removal of pathological metabolites (Zeng et al., 2021). In D-galactose-induced aging mice, gut microbial dysbiosis was reversed by Ganoderma triterpenoids through elevation of the abundance of Eubacterium lentum (E. lentum). Subsequent modulation of serine metabolism in the serum and brain was observed, by which cognitive dysfunction in aging model mice was effectively alleviated. The selective clearance of senescent cells is highly likely to be mediated by Ganoderma triterpenoid metabolites through the inhibition of NF-κB, TFEB, p38, ERK, and mTOR signaling pathways and the reduction of senescent cell burden, with apoptotic processes involved in this selective elimination.

To date, over 220 triterpenoid metabolites have been isolated and characterized from Ganoderma, among which Ganoderic acid A (GAA) emerges as the most abundant metabolite. GAA monomers are efficiently absorbed into systemic circulation and undergo enzymatic interconversion via reduction, oxidation, and desaturation pathways, ultimately being eliminated as more polar metabolites (Zhang et al., 2025). GAA is regarded as one of the most predominant bioactive metabolites of Ganoderma triterpenoids. Administration of GAA has been demonstrated to attenuate the accumulation of senescent cells and the deterioration of physiological function across multiple organs in radiation-induced premature aging mice, naturally aged mice, and Western diet-induced obese mice, with cellular senescence thereby being effectively ameliorated. GAA is widely recognized as a key metabolite mediating the anti-aging effects of the traditional Chinese medicine Ganoderma lucidum; however, the specific pharmacological mechanisms underlying the actions of GAA have not yet been fully clarified.

HT22 cells, which are derived from mouse hippocampal neurons, have been demonstrated to stably recapitulate the key pathological features of neuronal aging. These cells are characterized by simple and accessible culture conditions, can be readily maintained *in vitro*, and can be obtained in batches with uniform homogeneity. Accordingly, they can be employed for large-scale drug screening and mechanistic studies. HT22 cells are widely recognized as an optimal *in vitro* model for investigations into neurodegenerative aging mechanisms and drug screening. By means of thermal proteomics, alterations in protein thermal stability induced by drug binding can be directly detected under native cellular conditions, in the absence of exogenous tagging or protein overexpression. Endogenous drug targets can thus be screened in an unbiased manner at the whole-proteome level, and false-positive outcomes as well as biases associated with *in vitro* reconstitution systems in conventional target identification strategies can be effectively eliminated.

In the present investigation, an integrative omics-based strategy was implemented to systematically profile the direct molecular interactome of GAA in neuronal cells and construct a regulatory network map of the signaling cascades modulated by this triterpenoid. The findings from these experiments are expected to furnish mechanistic elucidation of the pharmacodynamic underpinnings of Ganoderma’s anti-aging properties and expedite the translational progression of precision therapeutic modalities.

## Materials and methods

### Mass spectrometry-based proteome thermal stability analysis

Sample Preparation and Protein Concentration Determination. HT22 cell samples stored at −80 °C were thawed on ice, subjected to ultrasonic lysis, and centrifuged to isolate supernatant protein solutions. Protein concentration was quantified using Bradford reagent, and the solutions were adjusted to a final concentration of 2 mg/mL for subsequent procedures.

Two groups of 550 µL samples were placed into 1.5 mL EP tubes. DMSO was added to one group, while 100 μM GAA (dissolved in DMSO) was added to the other. Each group was then gently mixed using a Vortex mixer, followed by rapid centrifugation. The mixed solutions of both groups were incubated at 25 °C for 1 h in the dark without agitation. After incubation, 10 aliquots from both the trl group (DMSO-treated) and the Compd group (GAA-treated) were transferred into PCR tubes. A temperature program comprising ten points (37 °C, 41 °C, 44 °C, 47 °C, 50 °C, 53 °C, 57 °C, 60 °C, 63 °C, 67 °C) was configured in a PCR machine. Samples were heated at each temperature for 3 min, rapidly transferred to ice, and centrifuged to harvest supernatants for downstream analysis.

Aliquots of the samples were transferred to 1.5-mL centrifuge tubes, mixed with Loading buffer, and denatured at 95 °C for 5 min. The denatured proteins were separated by 10% SDS-PAGE to assess thermal stability profiles. Samples were first treated with 1% SDS and 10 mMDTT, followed by incubation at room temperature in the dark for 30 min to reduce disulfide bonds. Subsequently, 25 mMIAA was added to alkylate cysteine residues, with the reaction proceeding in the dark for 30 min. A second treatment with 25 mMDTT was performed for 15 min at room temperature to quench excess IAA.

Carboxyl magnetic beads were added to the samples to precipitate proteins. The beads were resuspended and mixed at 37 °C with 1500 rpm agitation using a thermostatic mixer. Proteins were digested overnight with LysC and Trypsin. Following bead washing, supernatants were collected, dried under vacuum at 45 °C, desalted using C18 spin columns, and prepared for LC-MS/MS analysis.

### Mass spectrometry analysis

The dried peptide samples were resuspended in 0.1% (v/v) formic acid aqueous solution and prepared for LC-MS/MS analysis. A label-free data-independent acquisition (DIA) method was developed and optimized by dividing the full m/z range into consecutive windows and systematically acquiring MS2 spectra for all peptide precursor ions within each window. For method validation, a representative 37 °C sample from the DMSO control group was subjected to data-dependent acquisition (DDA) with a 60-min gradient injection. Subsequent database searching using Proteome Discoverer 2.4 was performed to determine optimal MS2 fragmentation windows for the entire proteome. Finally, proteomic samples were rehydrated and analyzed using the validated DIA method with a standardized 60-min gradient program on a Q Exactive HF-X mass spectrometer.

### DDA split window

Separation was conducted on an Easy nLC 1200 UHPLC system, coupled with a reversed-phase analytical column (25 cm in length, 75 μm inner diameter). The mobile phase was composed of Phase A (0.1% (v/v) formic acid in ultrapure water) and Phase B (0.1% (v/v) formic acid in 80% acetonitrile), and gradient elution was employed for chromatographic separation. The gradient program was specifically set as follows:

0–1.8 min: 1%–3% Phase B;

1.8–2 min: 3%-5% Phase B;

2–26 min: 5%–15% Phase B;

26–47 min: 15%–28% Phase B;

47–54.5 min: 28%–38% Phase B

Thereafter, the proportion of Phase B was rapidly ramped up to 100% within 0.1 min and maintained at this concentration for 10.4 min. A constant flow rate of 300 nL/min was maintained throughout the entire elution procedure.

Mass spectral data were acquired in positive ion mode using a mass spectrometer equipped with an Orbitrap mass analyzer. The mass spectrometry parameters were optimized as follows: full MS spectra were acquired over a mass range of 350–1800 Da at a resolution of 60,000 in profile mode; data-dependent acquisition (DDA) was utilized for MS^2^ spectral acquisition in centroid mode with a resolution of 15,000. Additional optimized parameters included an isolation window of 1.6 m/z units, a higher-energy collisional dissociation (HCD) collision energy of 28%, a maximum ion injection time (IT) of 50 m, and a total of 21 scans for the Full ms-ddMS^2^ acquisition method.

### DIA data acquisition: DIA method development based on DDA window partitioning

The Easy nLC1200 UPLC system was utilized with mobile phase A (0.1% (v/v) formic acid in water) and phase B (0.1% formic acid in 80% acetonitrile). Separation was performed on a reversed-phase analytical column (25 cm, 75 μm i.d.) using the following gradient program:

0–1.8 min: 1%–3% B

1.8–2 min: 3%–5% B

2–26 min: 5%–15% B

26–47 min: 15%–28% B

47–54.5 min: 28%–38% B

Thereafter, the proportion of Phase B was rapidly ramped up to 100% within 0.1 min and maintained at this concentration for 10.4 min. A constant flow rate of 300 nL/min was maintained throughout the entire elution procedure.

Mass spectral data were acquired in positive ion mode using a mass spectrometer equipped with an Orbitrap mass analyzer. The mass spectrometry parameters were optimized as follows: full MS spectra were acquired over a mass range of 350–1800 Da at a resolution of 60,000 in profile mode. Data-independent acquisition (DIA) of MS^2^ spectra was performed in centroid format with a resolution of 30,000; 32 variable isolation windows covering the full precursor m/z range; HCD Collision Energies, 28%. FDR: 1%.

### Bioinformatics analysis

Protein and Peptide Annotation: Melting curves for DMSO- and GAA-treated groups were generated using the Bioconductor TPP package, followed by nonlinear regression to compute half-melting temperatures (Tm). Proteins failing to meet curve-fitting quality thresholds (R^2^ < 0.8 in either group, control plateau ≥0.4, Peptide FDR <0.01, Protein FDR <0.01, and Missed cleavages = 2) were automatically filtered out via scripted quality control. Remaining proteins were processed using Spectronaut software for label-free quantification, integrating precursor ion intensities across thermal fractions. Thermal stability profiles were modeled for the top 20 proteins exhibiting the greatest differential melting behavior between conditions, with visualization performed using ggplot2 to generate melting curve overlays. Proteins displaying the most significant thermal shifts (ΔTm >3 °C, p < 0.05 by Welch’s t-test) were prioritized for downstream validation.

### Mass spectrometry data analysis and processing

Raw mass spectrometry data were processed using Spectronaut software with the following parameters (Table 1).

**TABLE 1:** Analysis parameters and settings.

Project	General parameters
Enzyme	Trypsin/P
Database	Uniport-mouse-filtered-reviewed
Fix modification	Carbamidomethyl [C]
Dynamic modification	Acetyl [ProteinN-term]; Oxidation [M]
Max missing cleavage sites	2
MS tolerance	10 ppm
Fragment mass tolerance	0.02 Da
FDR	1%

### Molecular docking

The 3D structural file of GAA was retrieved from the PubChem database (https://pubchem.ncbi.nlm.nih.gov/), and the CASTOR1 protein was acquired from the PDB database (https://www.rcsb.org/structure/5GT8). In the absence of known binding sites for the CASTOR1 protein, a molecular docking strategy was employed wherein the entire protein surface was designated as the candidate region, enabling the small-molecule GAA to explore the binding pocket with the optimal binding energy. The CASTOR1 protein was pretreated using PyMOL 2.3.4 software to remove water molecules and ligands, thereby eliminating interference from non-specific binding. Subsequent chemical modifications, including hydrogenation and charge equilibration, were conducted using AutoDockTools software. Both the receptor protein and the small-molecule ligand were individually converted to the PDBQT format. Molecular docking between the receptor protein and the small-molecule ligand was performed using AutoDock Vina 1.1.2, with the exhaustiveness of global search set to 32 and all other parameters retained at their default values. The docking results were analyzed via PLIP and visualized using PyMOL 2.5.5.

## Results

**FIGURE 1:**
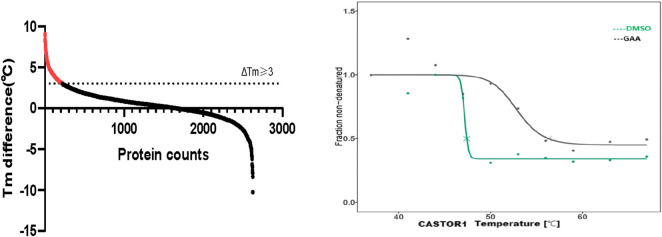
Left panel: The number of proteins exhibiting significant stability differences following binding to GAA. Right panel: Thermal melting curves of protein CASTOR1 showing differential stability profiles between GAA-treated and DMSO-treated samples.

Upon lysis of HT22 neuronal cells, total protein was extracted, incubated with GAA and subjected to thermal treatment across a gradient of temperatures. Following centrifugation to remove denatured proteins, the resultant supernatant was digested with trypsin, and the peptides were desalted prior to analysis via data-independent acquisition mass spectrometry (DIA-MS). This workflow yielded 88,197 precursor ions, 69,056 unique peptides, and 5,161 proteins, which underwent differential thermal stability analysis (Figure 1). A total of 5161 proteins were subjected to thermal proteome profiling analysis (TPP) and subsequent screening based on the following criteria: a melting point difference (ΔTm) ≥ 3 between the administration group and the control group, an R^2^ > 0.8 for both groups, and a plateau <0.4 in the control group. To eliminate false-positive results, database searching and analysis were conducted using Spectronaut software, with the following parameters configured: Peptide FDR <0.01, Protein FDR <0.01, and Missed cleavages = 2. Consequently, 220 proteins with significantly enhanced stability following GAA treatment were identified, and the results are presented in Figure 1. From this subset, the top ten proteins displaying the most pronounced shifts in melting curves (Nt5c, Cryab, Sumf1, Smad4, Castor1, Eif1a, Prpf38a, Rpl38, Mien1, and Nudt9) were selected for functional enrichment analysis (Table 2).

**TABLE 2:** The top 10 proteins with the most pronounced thermal stability differences and their annotated functions.

Number	PG.Genes	PG.Protein descriptions	Function
1	Nt5c	5′ (3′)-deoxyribonucleotidase, cytosolic type	Nucleotide metabolism, Energy metabolism (Moniz et al., 2017)
2	Cryab	crystallin alpha B	Molecular chaperone, Anti-apoptosis (Thorkelsson et al., 2025)
3	Sumf1	Sulfatase modifying factor 1	Activate sulfate esterase (Lin et al., 2024)
4	Smad4	Mothers against decapentaplegic homolog 4	The key transcription factors in the TGF-β signaling pathway (Sun et al., 2025)
5	Castor1	Cytosolic arginine sensor for mTORC1 subunit 1	Arginine sensor, The mTORC1 signaling pathway (Chen et al., 2025)
6	Eif1a	Eukaryotic translation initiation factor 1A	Translation initiation, ribosome assembly (Li L et al., 2019)
7	Prpf38a	Pre-mRNA-splicing factor 38A	Splice the precursor mRNA (Wang et al., 2017)
8	Rpl38	Large ribosomal subunit protein eL38	Ribosome assembly, Protein synthesis (Qin et al., 2023)
9	Mien1	Migration and invasion enhancer 1	Inhibit the migration and invasion of tumor cells (Xu et al., 2022)
10	Nudt9	Nudix hydrolase 9	The energy metabolism and signal transduction processes within cells (Debar et al., 2023)

**FIGURE 2:**
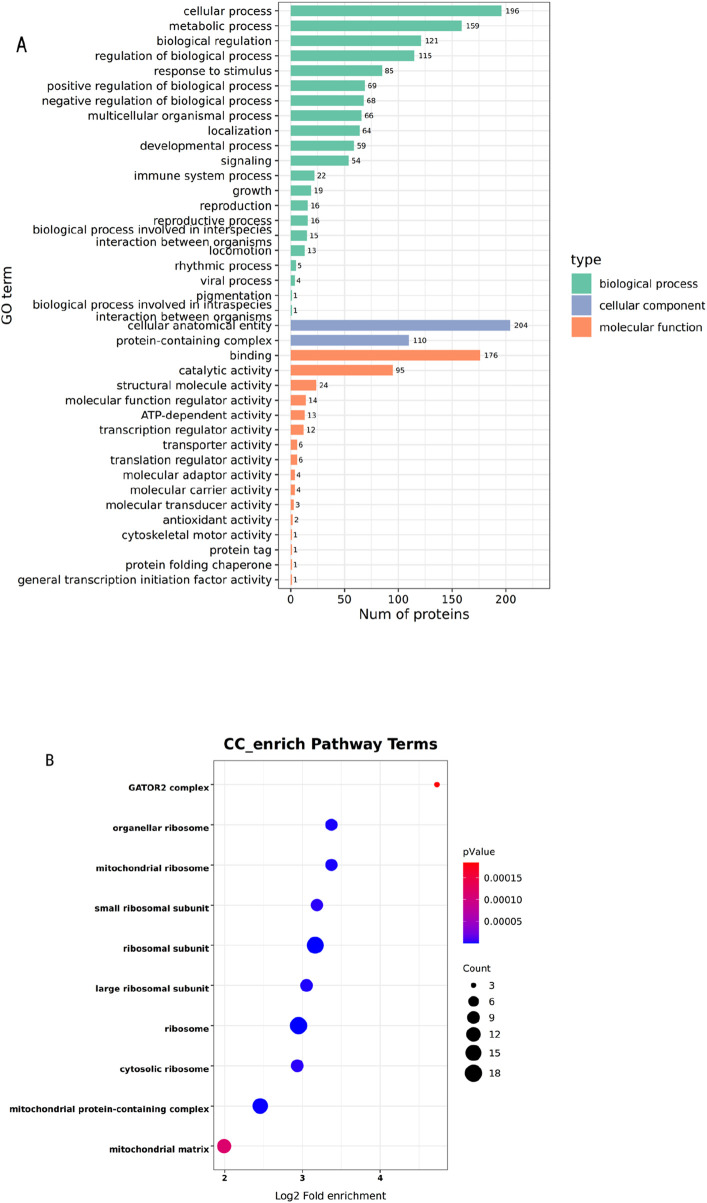
**(A)** GO annotation statistics **(A)** are presented for proteins exhibiting significant thermal stability differences. **(B)** GO enrichment analysis **(B)** are presented for proteins exhibiting significant thermal stability differences.

Subsequently, proteins underwent GO secondary functional annotation and enrichment analysis (Figure 2). The top 10 significantly enriched GO terms (p-value ≤0.05) were rendered as an enrichment bubble plot. Through statistical evaluation of GO term significance (p-value), significantly enriched functional categories and signaling pathways of GAA-bound proteins were delineated, and protein functional localization was computationally inferred. Results indicated that GAA-associated proteins were predominantly categorized into three ontological domains: Biological Process, Cellular metabolite and Molecular Function (Figure 2A). Among these, biological processes exhibited the highest enrichment significance, primarily encompassing cellular metabolic processes, metabolic regulation, and biological signaling. For cellular metabolites, pronounced enrichment was detected in cellular anatomical entities, whereas molecular function annotations were dominated by binding activities. Collectively, these data suggest that GAA modulates metabolic and regulatory networks through targeted interaction with specific molecular proteins.

GO enrichment analysis was performed, and the GATOR2 complex was identified as the most significantly enriched functional unit, with three binding proteins annotated. GAA displayed significant enrichment in the GATOR2 complex-associated functional category, highlighting a robust correlation between GAA and this complex (Figure 2B). In the detection of differences in protein thermal stability, a significant change in the stability of SEH1L protein—a downstream molecule of GATOR2—was observed within the tested temperature range. SEH1L protein is one of the core subunits of the GATOR2 complex and plays a crucial role in processes such as amino acid sensing by GATOR2 and regulation of the mTORC1 signaling pathway. CASTOR1 has been established by Valenstein et al. as a key target molecule in the arginine-mTOR signaling pathway (Valenstein et al., 2022). When arginine depletion leads to the activation of CASTOR1, the active form of CASTOR1 binds to GATOR2 and inhibits its function, thereby maintaining Rag GTPases in an inactive state (Yang et al., 2024; Chantranupong, et al., 2016). In cases where the release of GATOR2 is dysregulated, mTORC1 becomes persistently overactivated, which induces the accumulation of misfolded proteins and mitochondrial dysfunction (Li T et al., 2019; Lutt and Brunkard, 2022).

**FIGURE 3:**
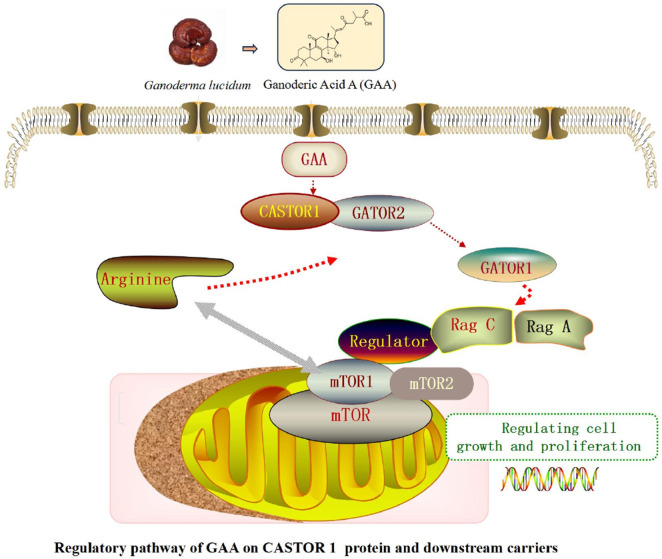
The signaling pathway diagram depicting the targeting of CASTOR1 by GAA, The red dashed line indicates an antagonistic effect.

GAA senses arginine levels and binds to the target protein CASTOR1. This binding event inhibits the excessive release of downstream GATOR2, ultimately regulating the activation of mTORC1. GAA has been experimentally linked to the mTOR pathway, with evidence showing its ability to ameliorate hypoxia-induced NSC damage and improve outcomes in Alzheimer’s disease mouse models via mTOR activation (Shen et al., 2021). Notably, the precise molecular mechanisms through which GAA interacts with nodal proteins to regulate mTOR signaling remain to be elucidated (Figure 3).

To characterize the enrichment patterns of metabolite-binding proteins in KEGG pathways, Fisher’s exact test was applied to derive their KEGG pathway enrichment profiles. Statistical significance of pathway enrichment (p-value) was evaluated to identify significantly enriched KEGG pathways. The top significantly enriched KEGG pathways (p-value ≤0.05) were selected for graphical representation as enrichment bubble plots. Additionally, protein sequences of differentially expressed proteins underwent structural domain prediction via the InterProScan integrated platform. Prediction outcomes were subjected to statistical summarization, and the top 10 most frequently occurring domains in differentially expressed proteins were visualized using heatmaps or bar charts (method specified based on data characteristics).

**FIGURE 4:**
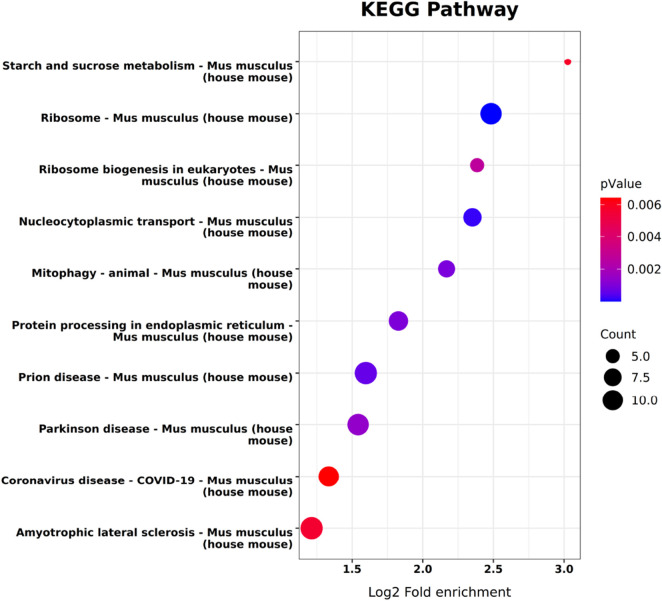
KEGG pathway enrichment analysis of binding proteins.

**FIGURE 5:**
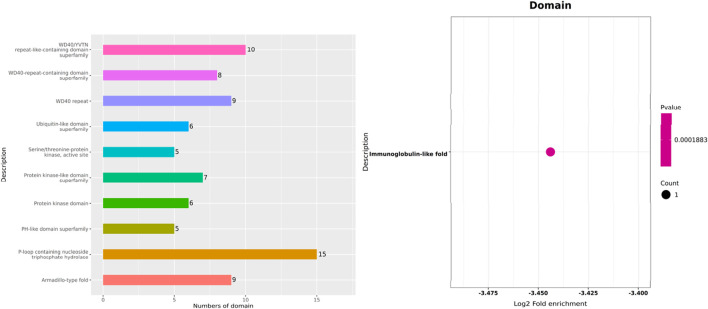
Protein domain enrichment analysis.

KEGG pathway analysis has demonstrated that GAA is predominantly engaged in starch and sucrose metabolism. Protein domain analysis has identified a significant association of GAA with the “P-loop containing nucleosidetriphosphate hydrolase” functional category (Figures 4, 5). This category is classified under the NTPase superfamily, which is primarily tasked with hydrolyzing nucleoside triphosphate (NTP) into nucleoside diphosphate (NDP) and pyrophosphate (PPi). Collectively, these results support the involvement of GAA in energy metabolism, signal transduction, cell division, and DNA replication.

**FIGURE 6:**
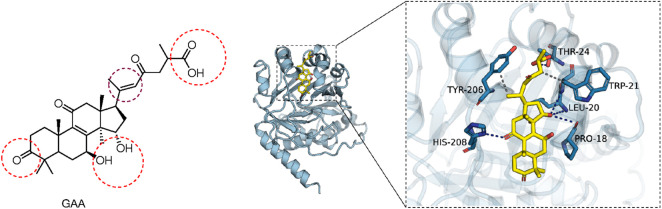
Active sites of ganoderic acid A (left); molecular docking simulation of ganoderic acid A with CASTOR1 target (right).

To elucidate the binding mechanism between GAA and CASTOR1, molecular docking was utilized. The active sites of GAA have been confirmed to be predominantly composed of carboxyl, hydroxyl, and acetyl groups (Sharma et al., 2019). Molecular docking (Figure 6) and molecular dynamics simulations were conducted for GAA and CASTOR1. Docking outcomes revealed that ganoderic acid A forms hydrogen bonds with PRO-18, LEU-20, TRP-21, THR-24, and HIS-208 residues in CASTOR1, thereby stabilizing the protein-small molecule complex. Furthermore, hydrophobic interactions between the small molecule and TRP-21/TYR-206 residues are responsible for providing strong van der Waals forces, contributing to the robust binding affinity of the molecular complex.

The active groups of GAA documented in the literature (Sui et al., 2025; Ali et al., 2025) are primarily the carboxyl group (-COOH) at C-26, the carbonyl group (-C=O) at C-11, the double bond (-C=C) at C-24, and the hydroxyl groups (-OH) at C-7 and C-15. These active groups were consistent with those observed in the present molecular docking (red circled region), thus validating the reliability of the molecular docking results.

## Conclusion

In this study, 220 proteins with significantly enhanced stability upon binding to GAA were screened using thermal proteome profiling. The top five proteins exhibiting distinct differences in melting curves were identified as Nt5c, Cryab, Sumf1, Smad4, and Castor1. Among the top 10 targets exhibiting significantly altered thermal stability identified by thermal proteomic profiling, numerous candidate targets were observed to be closely associated with diverse aging-related mechanisms. Smad4 is recognized as a key regulator of the TGF-β signaling pathway involved in cellular growth and immune modulation (Liu et al., 2025). Notably, Smad3, a downstream signaling molecule of Smad4, has been extensively reported to participate in multiple critical processes during cellular senescence. These processes are known to encompass telomere shortening, epigenetic dysregulation, autophagic dysfunction, mitochondrial impairment, chronic inflammation, extracellular matrix remodeling, and metabolic disturbance. Cellular senescence can be triggered by the TGF-β/Smad3 signaling axis, an effect considered to be mediated by the regulation of autophagic flux (Yong et al., 2020). Furthermore, multiple additional targets, including Cryab, have been confirmed to be closely implicated in aging-related pathways (Margaret et al., 2023).

Furthermore, multiple strategies such as GO enrichment analysis and KEGG analysis were utilized to identify the most central potential core targets. In the TPP assay, markedly altered thermal stability was also observed for SEH1L, which functions as a downstream protein of the CASTOR1 receptor. GO enrichment analysis of the 220 proteins revealed that the highest enrichment of GAA was observed in the downstream GATOR2 complex of CASTOR1. CASTOR1 homodimers are known to interact with the GATOR2 complex for arginine metabolism regulation (Sah et al., 2021). Upon arginine deprivation, dimerization between CASTOR1 and GATOR2 is induced, which further promotes the conversion of RagA to its GTP-bound form and RagC to its GDP-bound form at the lysosomal membrane (Yang et al., 2024). These events subsequently modulate the Ragulator complex, which is regarded as a key scaffold responsible for anchoring mTOR to the lysosomal membrane, by which the mTOR signaling pathway is regulated.

A close association between GAA and the mTOR pathway has been experimentally confirmed. It has been proposed that GAA may ameliorate hypoxia-induced injury in NSCs through the activation of the mTOR pathway, with symptoms in mouse models of Alzheimer’s disease consequently being alleviated. Through KEGG pathway analysis, protein domain prediction, and molecular docking simulation, CASTOR1 is ultimately hypothesized to be one of the core targets of GAA in neural cells. It is speculated that GAA may exert its pharmacological effects by sensing arginine levels and mediating the mTOR signaling pathway. Experimental validation of this signaling mechanism remains necessary through cellular and animal model studies.

From a traditional Chinese medicine perspective, this research is aimed at identifying the core anti-aging pharmacometabolites of Ganoderma lucidum and elucidating the molecular mechanisms of GAA. The regulatory roles of Ganoderma lucidum in physiological function modulation, immune enhancement, and antitumor activity are contributed to be uncovered by the findings, thereby providing theoretical foundations for its clinical applications in disease prevention and treatment. Furthermore, new frontiers for the innovative development of Ganoderma lucidum-based therapeutic strategies are opened by this study.

## Data Availability

The data presented in the study are deposited in the iProX repository (https://www.iprox.cn/), accession number PXD071098.
